# Cholera and Calomel

**Published:** 1866-10

**Authors:** 


// HALL'S JOURNAL OF HEALTH.

OUR LEGITIMATE SCOPE IS ALMOST BOUNDLESS: FOR WHATEVER BEGETS PLEASURABLE AND HARMLESS FEELINGS, PROMOTES HEALTH; AND WHATEVER INDUCES DISAGREEABLE SENSATIONS, ENGENDERS DISEASE.

*We aim to show how Disease may be avoided, and that it is best, when sickness comes, to take no Medicine without consulting an educated Physician.*

VOL. XIII.] OCTOBER, 1866. [NO. 10

### CHOLERA AND CALOMEL.

CONFIRMATION of our January article, written in 1854, is found in the following, which has just made its appearance, and of which and its author, the New York *Evening Post* says—

"We find in the current number of the Cincinnati *Lancet and Observer* an article by Dr. John Davis, one of the most eminent and skillful allopathic physicians of the western states, in which he gives particulars concerning the treatment of cholera, which will, doubtless, have interest for those of our readers who adhere to the allopathic method.

After reviewing the different modes of treatment and re medies adopted in various countries, and showing their uncertainty, Dr. Davis says:

### SYMPTOMS.

'I judge, therefore, that, as yet, we are safest in seeking in the symptoms of this disease for guides for its m anagement.—Examining the course of the malady, it is very often found that its attacks are preceded by laxity of the bowels corresponding to ordinary diarrhœa, the stools not being white nor rice-water in character. We are not warranted in pronouncing such cases cholera; for during the epidemic prevalence of this pestilence, perhaps a large part of the population are thus troubled, and though many of them take no medicine, they escape any serious illness. This kind of diarrhœa the French have named cholerine; but inasmuch as Dr. Farr has applied this word as a term for the zymotic cause of cholera, it is less likely to cause confusion if we confine ourselves to the use of the old name diarrhœa for this condition. The indications for its treatment are simply to use ordinary astringents.

'When, however, the discharges assume the rice-water form, or are white and copious, we are warranted in concluding that cholera is present. Dr. Drake, in one of his letters to the public, in the early part of 1849, very emphatically declared, that when this character of stool appears, it is just as sure that cholera is present as that your house is on fire when as yet only a few shingles are burning. Yet the course taken by many regular physicians in this city was to consider nothing as cholera that did not run through all the stages of the disease, including collapse, and often even death. When a city ordinance required a statement for the public from each practitioner of the number treated and the results, one estimable and excellent physician, holding the views to which I have referred, reported four cases and four deaths. So strong was the disposition among regular physicians to attach odium to any report of success at that time, that I declined making any return concerning my own cases.

'Rice-water diarrhœa, or diarrhœa presenting copious whitish stools, appearing when cholera is epidemic, being regarded by all of the writers as sufficiently evincing the presence of the malady, it is our duty to acquiesce in this conclusion.

'These discharges manifest the absence of bile, and that we need something that will cause this secretion to flow into the intestines, and for this purpose no agent is so powerful as calomel. And our experience teaches us that even in common diarrhœa attended with whitish discharges, and particularly in the case of young children having this kind of evacuations, we are very slow and uncertain in arriving at success, except when we combine a limited amount of this agent with our other means.

'Another indication is to control the diarrhœa by the administration of astringents in company with the calomel.

'Pursuing our investigations, we find that an essential feature of the disease is the more or less rapid failure of the capillary circulation, and to counteract this tendency no medicines are so effective as piperine and capsicum. They determine more to the surface than any other stimulants that have not otherwise a mischievous action in the condition of a cholera patient. So active are they, that a well person taking a full dose of either is hot all over, often in a few minutes.

'As the attack advances, vomiting, intense thirst and suppression of the urine occur, accompanied with violent cramps in the limbs; and if the attack is not controlled, collapse and death follow.

'Such is the general description of this disease, but the cases considered individually often present minor difficulties which require attention almost as much as the graver symptoms.

TREATMENT.

'My course upon meeting with a case of Asiatic cholera of the ordinary form was to administer something amounting to the following, viz: Calomel, ten grains: gum kino, twenty grains; piperine, ten grains; prepared chalk, one drachm. Mix and divide into ten parts. One of these powders to be given every ten minutes, or even only every three hours, according to the condition of the patient.

'Instead of this formula I frequently used the following, viz: Blue mass, one scruple; tannin, two scruples; piperine, one scruple. Mix and make into twenty pills. One of these to be taken every twenty minutes, or only one every two or three hours.

'For the vomiting I ordered mustard poultices over the stomach, and when this did not suffice I prescribed the following preperation for internal use, viz: Creosote, half a drop; chloroform, from half a fluid drachm to a fluid drachm; simple syrup, half a fluid ounce; peppermint water, one and a half fluid ounce. Mix. A teaspoonful of this to be given every ten or twenty minutes while the vomiting continued.

'Notwithstanding the intense thirst, I forbade the use of water except in tablespoonful measures sparingly supplied, having found that in larger quantities it was immediately vomited. I however allowed small pieces of ice to be kept in the mouth; and I gave water liberally after the vomiting had ceased.

'As to the mercury in the foregoing prescription, I discontinued its use as soon as the stools were darkened in color, or the diarrhœa was arrested. My observations led me to conclude that the further administration of mercury tended to the establishment of dysentery and attending fever. When the diarrhœa persisted and the discharges were of a darkened color, omitting the calomel or blue mass, I continued the other parts of the treatment; and when the diarrhœa was checked, I left off the astringents, continuing the use of the stimulant till reaction was fully re-established, and often combining a grain of quinine with each dose of the stimulant. In some cases I used Huxham's tincture of bark, or some preperation of iron, instead of the quinine, as a tonic.

'Soon after the appearance of reaction the kidneys usually resume the performance of their function; and there was seldom an occasion for a resort to diuretics. The plenteous supply of water, administered as soon as the patient was able to retain it, almost always sufficed.

'This was the general plan of treatment. For the lesser troubles that often attended I ordered as the circumstances seemed to demand. Opium or brandy I did not prescribe, except in special cases; and then only when the attack was in an early stage, owing to the fact of what I saw myself and the testimony of some of the highest authorities, that their use tends to increase the danger of the occurrence of fever and cerebral difficulties when the patient has survived the first stage. Blistering with cantharides over the epigastrium was in 1832 extensively practiced in Europe; but the little benefit of it was so manifest that no systematic writer now recommends it.

'Frictions I discountenanced, inasmuch as they increase the alarm of the sick and excite the minds of the attendants, without producing any benefits adequate to compensate for their evil effects. And this course I also pursued with everything else that was likely to add to the fears of the sufferer.

'I wish I were able to state the proportion of recoveries under the plan which I pursued; but I know recovery was so frequent that even when called to a severe case I expected the patient to get well. Even a large number of my collapsed cases survived. I may add that the great majority of my patients got well in a very few days without passing through a stage of fever, or having any cerebral disturbance.

'My field of observation during the invasion of 1849 was extensive, as many of you know. My office was in the crowded German portion of our city, where every physician had more calls to visit persons stricken with this disease than, even with the utmost taxing of his physical powers, he was able to attend.'"

——As a matter of general interest we append the following

Cholera prescription. — The Board of Health of New York city recommends the following prescription in severe cases of Diarrhœa, when the services of a physician can not be immediately obtained, "Tincture of Opium, Tinctureof Camphor, Tincture of Capsicum, of each one drachm; Chloroform, half a drachm; mix and take half a teaspoonful after each evacuation. This will in most instances cure a diarrhœa, but it does not follow that it will cure Cholera. The typhoid symptoms which the disease leaves behind it in the system must have other treatment; but much has been gained when these characteristics have been overcome. By the use of the above remedy until a patient can be seen by a physician, a recovery may occur which would be hopeless if the disease were permitted to go unchecked into a full or even partial collapse."

As Diarrhœa, Dysentery and Cholera are in the same class of diseases, and when cholera prevails there is more or less of the other two present at the same time and are liable to run into cholera, we advise all our readers to have a three ounce vial of the following in their houses in case of an attack in the night or its being impossible from any cause to secure promptly the services of a physician. The very fact of giving something which is recommended by such high medical authority has of itself a quieting and soothing influence on the patient in promoting a recovery; but bear in mind always that Dysentery gives bloody discharges with much distressing and unavailing straining. Diarrhœa gives large, thin, yellow, green or dark, ill-smelling discharges with more or less griping. Cholera gives neither griping nor blood, but a copious, forcible, whitish, thin, inodorous, painless and most exhausting discharges; one of which is cholera begun, to be followed up by others, bringing on in a few hours vomiting, cramps, suppression of urine, hoarse voice, blue skin covered with large drops of sweat, and death, with generally a calm, composed and perfectly rational mind to the very last.

### TREATMENT OF DYSENTERY.

The Nashville (Tenn.) *Journal of Medicine and Surgery* contains an article on the above subject, by J. W. Brown, M. D., the substance of which will be of interest to many of our readers. He states that dysentery is the principal disease with which the physician has to contend in Tennessee, Arkansas and North Louisiana, and in some localities the mortality is frightful. Drs. McMath and Weilder, of Louisville, Ark., informed him that they had treated three hundred cases of the most aggravated form with success by the use of creosote, and in every case in which it was given (if not delayed too long,) a marked improvement took place.

The following is the formula used by these gentlemen:

Capsicum, 10 drops; acetic acid, 20 drops; sulphate of morphine, 2 grains—all mixed in an ounce of distilled water. A teaspoonful of this is given every three or four hours to adults; smaller doses are given to children, in gum arabic mucilage. Drs. Mc Math and Weilder consider it nearly, if not quite, a specific in dysentery.

This disease is sometimes very fatal and prevalent in all parts of our country, and children about two years old, in the cities, are very liable to be attacked with it in the months of July, August and September. Creosote and morphine alone, we understand, are given in such cases by our New York physicians, but with what general success we can not tell."

Whole volumes could be written about cholera, but there are two points upon which every observant reader should keep his attention steadily fixed, that is, the actual facts connected with its exciting cause and cure. The original cause we know but little about, but it must be a something which precedes the exciting cause and without which there can be no epidemic cholera; this is most certainly the fact that many things will excite an attack of cholera, when the disease is prevalent, which do not do so when it is not. But this original cause cannot produce a single case of the disease without the aid of an exciting cause; these two causes, the original and the exciting, are like powder and fire, harmless, unless brought in contact. Filth of person, clothing, dwelling, and locality, are regarded as among the indisputable, exciting causes; it is true only in part perhaps; not all kinds of filth cause cholera; the filth of decaying vegetation excites the disease in a community; but we have seen no proof that proximity to a grave-yard, to slaughter-houses, or bone-boiling, or glue establishments is more dangerous in that direction. A man in ordinary health may sleep in a slaughter-house for a month, in a cholera locality, and will not take the disease; if, other things being equal, he sleeps in a house on the windward side of a drained mill-pond, its muddy bottom exposed to a hot mid-day sun, any time from June to September, he will die in a week, if not in twenty-four hours. Whatever of a vegetable nature that is allowed to decompose by being kept moist and exposed to a hot sun, or the heat of a ship's hold in warm weather, will excite cholera, always and under all circumstances, where cholera is prevalent; when it is not prevalent, it will always cause diarrhœa, dysentery, and all forms of spring, and fall fevers, according to the degree of decomposition, from slight fever and ague which continues for months, and leaves the system as well as ever, to the congestive chill, which kills in a night. When any one thing causes epidemic cholera, it is important to know what that one thing is, so as to direct all the energies to its removal, instead of wasting them in the removal of other things comparatively harmless. No standing water on an earthen bottom ought to be allowed within a mile of any residence in cholera times; and in communities, every street gutter should be kept as clean as a broom can make it, or as dry as a powder-horn. Street filth, cellar filth, rear-yard filth, kitchen filth, all involving the decay of vegetable substances, these are they which make cholera victims in cities.

As to the pathology of the disease, we think it is the failure of the liver to withdraw the bile from the blood, it is torpid or relaxed, and does not do its legitimate work with proper activity; whatever restores this action, cures the case. All physicians of legitimate medicine, of all lands, know that calomel is the most certain medicine known to man, by a thousand-fold to stimulate the liver to its natural action; there are various other medicines which have a similar effect, but they can never be relied on, and when the time lost in a failure is literally death, in this terrible malady, surely the certain should be employed in place of the uncertain: besides this there is another incalculable advantage in calomel as in the progress of cholera in each case there is such an irrepressible vomiting, that even cold water is ejected with great force, the instant it reaches the stomach, much more, nauseating drugs, but calomel is so heavy that it sinks to the bottom of the stomach, especially if in the form of a pill, and is, in that form at least, impossible of ejection, and goes on to do its proper work, first of arresting the passages within two hours, next of changing their color.

Dr. Edward B. Stevens, the efficient and able editor of the Cincinnati Lancet and Observer, says that in the recent fearful ravages of the disease in Cincinnati, Ohio, the majority of the best physicians there have found the main reliance to be small doses of calomel, two or three grains every fifteen minutes, in combination, some with one thing, others with another, capsicum, piperine, opium, &c., while others, for whom he has a great esteem, give from ten to twenty grains at a time, and then rest, and wait for the result; this is precisely the course we hinted twelve years ago, and repeated in the last January number. Dr. Davis says, "his experiences this summer quite fully confirm the views advanced" in the article which we have just given our readers. We think the patient's life is endangered and time lost in feeling along with two or three grain doses at fifteen minutes interval, trying, as it were, to get along with the least amount of calomel possible, when in many cases a great deal more will be given in the aggregate, than the single large dose, to say nothing of the incessant disturbing of the patient whose inmost heart in all cases yearns to be let alone: this constant doing something for a cholera patient, plasters, frictions, dosing and inquiries, exhaust his strength, and impress him with a sense of danger which in many cases kick the beam in favor of the grave.

If the dejections of cholera patients cause cholera, and if it is spread by intercommunication, through travellers, it is difficult to explain its leaping during 1866, from the Atlantic coast to the Mississippi valley, requiring three months in the transit, while travellers perform the journey in three days, but on the malarial theory, vegetable decomposition, the explanation is easy and conclusive; the cholera has raged in the Western cities which are either built upon made ground or are in the immediate vicinity of a soil composed almost wholly of the remnant of vegetable decay, as in Cincinnati, St. Louis, Memphis, and New Orleans. If the fatality in New York had been equal to that of Cincinnati, we should have reached four hundred deaths in a day, instead of seventy-four, at its highest.

All know that the disease has waxed and waned in New York city thus far, in proportion to the heat of the weather, which causes vegetable decomposition, that is of kitchen offal, rinds, peelings, tops, decayed parts, dust; and as to the streets, weeds, grass, bits of wood, droppings of animals, made up wholly of vegetable matter. We do not object to putting away choleraic dejections, instantly and perfectly, as a matter of comfort, cleanliness, and decency, but the chief attention should be the removal or prevention of such filth as has been so much insisted on. Epidemic cholera is impossible under any circumstances in a pure air, or in a clean sandy plain, or in rocky mountain sides, because there is no vegetation there to decay.

With very great interest and satisfaction a letter from Dr. Davis is appended, this moment received, and he will excuse the liberty taken in publishing it, for he is known to be too great a lover of scientific truth to have any delicacy in the use of anything from his pen which would add to the knowledge of the profession or promote the public welfare.

CINCINNATI, Aug. 30th, 1866.

DR. HALL.

DEAR SIR.—In your little note under date of August 28th, you inquire whether my plan of treatment of Asiatic Cholera has been as successful in 1866 as it was seventeen years before?

I reply that it has;—that in every case but two, at most, in my practice, where the sick have been placed under it early, and the course strictly followed, recovery has been the result, and that without, in a single instance, the occurrence of consecutive fever.

It is a gratification to me to be also able to state that as many of the other physicians here as I have known to strictly adopt my course have been equally successful.

One of these, who probably has had as great a number of cholera patients as any physician in this city, during the epidemic of this summer, remarked to me to-day, "that pursuing this plan of treatment he has no fears for a cholera patient if before being called to attend, no opium or camphor has been administered, and the fluids of the body of the patient were not already almost completely drained away.

I have taken notes of every case of cholera of which I have had charge, and as soon as I have time I shall prepare a careful report from them.

My views of the disease have not changed in the least. Cholera as it has prevailed in this city during the last few weeks, has been as the books have described it everywhere. If there has been any difference between the cases of 1849 and those of the present year, the difference has been that the cases this year have been more persistent, and required the longer continuance of the administration of calomel before the discharges were darkened in color, or the diarrhœa lessened in violence.

Our city during the invasion which began in 1859, with half the population which it now has, lost four or five thousand people by cholera. This year we have lost only thirteen hundred. What next summer will show we cannot tell.

In looking over the names of those who have died here of the disease, as they have been published in the daily papers, the striking fact is manifest that they were almost every one either German or Irish; that is of the number who live in tenement houses, inhaling, of necessity, concentrated crowd poison during much of their time.

Yours respectfully and truly,

JOHN DAVIS,
323 Elm Street, Cincinnati.

We are not particularly anxious that our views of the nature, cause and cure of epidemic cholera should be adopted, but we do desire that all the real facts should be presented to the profession, and to intelligent general readers to aid in arriving at the true principles of practice.

While all the facts are fresh in the memory, the Board of Health of New York would do well to answer the following questions in the light of all their observations.

1st.—Were there a greater or less number of cholera cases in the vicinity of slaughter-houses, gas-works, glue manufactories, and bone-boiling establishments, than elsewhere?

2d.—If cholera dejections are a fruitful cause of spreading the disease, which seems to be becoming the almost general sentiment, must we suppose that only drunken persons and those living in low, flat, damp, or otherwise filthy localities, chanced to fall within the influence of those dejections?

3d.—Did not the heighth of the thermometer indicate the ravages of the disease?

4th.—Were not those ravages always greater in low, flat, damp localities, and were not high grounds and clean sections wholly exempt?

5th.—If the cholera is a portable disease, that is, carried by travellers from one part to another along the great lines of travel, as was so confidently and pertinaciously asserted, a few months ago, why has it not reached Boston and Albany before this? Why did it require three months to reach Philadelphia, less than ninety miles away; why has it passed the Alleghanies and leaped over Pittsburgh and lighted on Cincinnati, at the end of several months, when there is an incessant stream of travel of thousands of people daily from New York? It required several months for the disease to reach Harlem flats from the bay of New York, a distance of less than ten miles, travelled by thousands every day, in crowded cars. The disease appeared sooner in the North than in the warmer localities west and south, as Cincinnati and Charleston, because made lands are lower and more moist, and it required a more protracted heat to evaporate the water and leave the flat surfaces exposed to the sun. Boston and Albany were too cold or stony, Pittsburgh too precipitous to hold water. We think, therefore, that the malarial theory explains all these points more satisfactorily than any other.

The noisome establishments above named ought not to be allowed to remain one hour within the limits of any community. Their very presence has a pernicious moral effect, as well as physical; it is only contended that there is not sufficient proof to show that they develope cholera, in a cholera atmosphere.—What has been said, relates only to epidemic cholera, that which involves a number of cases, those which occur singly, from personal, individual, or transient causes, are not to be taken into account; we conclude, therefore, that the law of the spread of epidemic cholera is heat, causing vegetable decomposition in a cholera atmosphere.

---

Mad-Stone.—John Smith, of New Harmon, Ind., says that while living with the Indians, he learned that the Mad-stone was obtained from the Rennett of the Deer, and that he has one of them which will cure a bee-sting within the time one can hold his breath. Webster defines the Rennett, or Runnett, the prepared stomach or concreted milk found in the stomach of a sucking quadruped.

Rancid Butter is said to be restored to nearly its former sweetness, if three pounds are churned with half a gallon of sweet milk.

Musquitoes are said to be driven from a room if a piece of gum camphor, as large as the quarter of a hen's egg is placed on a piece of tin and held over a lamp, but not so near as to take fire. The London "Field" says that the branch of a walnut tree, suspended over a bed, is a good protection against gnats and musquitoes. The Editor will be obliged to any one who will make the experiment thoroughly, and report.

